# Navigating the Clinical Landscape of Liposomal Therapeutics in Cancer Treatment

**DOI:** 10.3390/pharmaceutics17020276

**Published:** 2025-02-18

**Authors:** Andreja Kozak, Ernestina Lavrih, Georgy Mikhaylov, Boris Turk, Olga Vasiljeva

**Affiliations:** 1 Department of Biochemistry and Molecular and Structural Biology, Jožef Stefan Institute, 1000 Ljubljana, Slovenia; andreja.kozak@ijs.si (A.K.); ernestina.lavrih@ijs.si (E.L.); georgy.mikhaylov@ijs.si (G.M.); boris.turk@ijs.si (B.T.); 2 Jožef Stefan International Postgraduate School, 1000 Ljubljana, Slovenia; 3 Faculty of Chemistry and Chemical Technology, University of Ljubljana, 1000 Ljubljana, Slovenia

**Keywords:** liposomes, drug delivery, cancer treatment, controlled drug release, clinical trials, clinical applications

## Abstract

Liposome-based targeted drug delivery systems represent a significant advancement in pharmaceutical science, offering distinct advantages that enhance the efficacy and safety of various therapies. These versatile carriers can encapsulate both hydrophilic and hydrophobic drugs, making them particularly valuable in clinical settings. This review explores the critical role of liposomal formulations in improving drug pharmacokinetics and minimizing side effects, especially in oncology, where targeted delivery to tumor cells is essential. Outlining the properties of different types of liposomes, we focus on the effects of these properties on the liposomes’ targeting and drug release capabilities through innovative surface modifications and describe the most common methods of liposome preparation and characterization. Furthermore, this review provides an in-depth analysis of the properties and composition of liposomal-based nanocarriers, with a unique focus on ongoing clinical trials and recently approved therapies. It offers a comprehensive overview of the latest advancements in pre-clinical research and highlights the critical progress in clinical development, offering insights into the clinical impact and regulatory approvals. Ultimately, this review underscores the transformative potential of liposomal nanocarriers in modern therapeutics, suggesting avenues for future innovations and clinical breakthroughs.

## 1. Introduction

Efficient drug delivery to tumors is one of the key challenges in cancer treatment that often limits therapeutic success and patient outcomes. Drug delivery systems have the potential to improve the therapeutic index by increasing drug concentration and residence time, and by minimizing side effects [1]. These systems have to overcome multiple challenges in the tumor microenvironment (TME), such as dense stroma or increased interstitial fluid pressure keeping the therapeutic agents from reaching their target. Low bioavailability due to fast elimination from circulation or biotransformation is a frequently encountered obstacle that can be significantly improved with an efficient delivery system [2].

Drug delivery nanocarriers include a plethora of different types of nanoparticles: lipid-based vesicles, such as micelles or liposomes; metallic nanoparticles, including nanoshells and quantum dots; many different types of polymeric systems, such as dendrimers; viral nanocarriers; carbon nanotubes; and others [3]. However, not many of these systems have been approved by the FDA, while more are in clinical trials [4]. Among the most promising and most explored systems are liposomes—spherical lipid vesicles composed of one or more lipid bilayers [5]. Liposomes were first described by Alec Bangham in 1961 and have since then become the most widely used nanocarrier in targeted drug delivery [6]. Their success is due to their convenient characteristics: high biocompatibility, biodegradability, easy functionalization, low toxicity, and immunogenicity. In addition, they offer the option to encapsulate hydrophilic drugs into their aqueous interior, while lipophilic compounds can be incorporated into the lipid bilayer [5].

Liposomal drug delivery systems have been successfully translated into clinical settings. The first FDA-approved drug was Doxil^®^, liposomal doxorubicin used to treat ovarian cancer, introduced in 1995 [7]. In the following years, more liposomal cancer treatments were developed and, to a lesser degree, some liposomal formulations for other diseases such as fungal infections. Liposomes have also become an important carrier system for vaccine development, such as vaccines for hepatitis and influenza [8]. More recently, lipid nanoparticles, a delivery system based on liposomes, were used in the development of mRNA vaccines for COVID-19 [9].

This review will focus on the clinical applications of liposomal drug delivery systems in cancer treatment, highlighting their advantages and limitations in real-world settings, as well as exploring future clinical avenues for their optimization and broader therapeutic impact.

## 2. Liposomal Drug Delivery Systems

Liposomes are nanosized lipid spheres, composed of lipid bilayers, as opposed to the related nanostructures of micelles, which contain a lipid monolayer. Based on their size and number of bilayers, liposomes can be classified into distinct categories: multilamellar vesicles (MLVs) contain several bilayers and are usually larger than 500 nm in diameter; unilamellar vesicles are composed of one lipid bilayer and can be further categorized as large unilamellar vesicles (LUVs), which are bigger than 100 nm in diameter, and small unilamellar vesicles (SUVs), with diameters of less than 100 nm. Another class of liposomes is multivesicular vesicles (MVVs), which contain many unilamellar vesicles within larger liposomes. MVVs range in size from 1 to 100 µm in diameter. The size of the vesicles is an important factor in determining the system’s circulation half-life and uptake, as well as its encapsulation efficiency. The liposomes employed in drug delivery systems are most often unilamellar and range in size from 50 to 150 nm [10].

The lipid bilayer significantly impacts many characteristics of the liposomes, which are crucial for their efficacy as drug delivery vehicles. Liposomal membranes are comprised mainly of phospholipids, which can be natural or synthetic; in general, liposomes comprised of natural phospholipids are less stable than those of synthetic. Lipid composition highly affects the characteristics of liposomes, such as particle size, rigidity, fluidity, stability, and electrical charge [11]. A phospholipid molecule is bipolar, composed of hydrophobic fatty acyl chains, a glycerol or sphingosine backbone, and a hydrophilic headgroup. Another crucial component of the bilayer is steroids—the major steroid used in liposome preparation is cholesterol, which improves liposomal rigidity and stability [12].

### 2.1. Types of Liposomes

Conventional liposomes, or liposomes of the first generation, were comprised only of natural or synthetic phospholipids [13]. Very soon, cholesterol was added to improve liposome membrane rigidity. These liposomes showed excellent tumor penetration and anti-tumor activity; however, their blood circulation time was low due to elimination by the mononuclear phagocyte system, and they have shown limited stability in vitro [14].

In comparison, charged liposomes are more stable during storage as they repel each other and are thus less prone to aggregation. Cationic liposomes are used in gene therapy due to their ability to encapsulate nucleic acids [15]. They are also considered as potential tools to deliver drugs to the brain, since they can cross the blood–brain barrier [16]. Anionic liposomes are less stable in the bloodstream compared to neutral and cationic liposomes; however, they can be used for transdermal drug delivery as they have been shown to improve penetration properties through the skin [17].

Second-generation liposomes were developed to tackle the problem of rapid clearance from circulation. Polyethylene glycol was added to the surface of the liposomes to increase their steric protection and to inhibit their recognition by the mononuclear phagocyte system. The PEGylated liposomes were named ‘stealth’ liposomes and showed improved circulation time and better target accumulation compared to conventional liposomes [18,19]. Stealth liposomes accumulate in the target tissue by passive targeting. This approach takes advantage of the enhanced permeability and retention (EPR) effect of the vasculature, brought on by abnormal leaky blood vessels and the lack of functional lymphatics in the tumor tissue [20]. This effect is aided by the increased blood circulation time of the stealth liposomes; however, the EPR effect alone cannot completely attenuate the side effects of cytotoxic drugs. Additionally, it is limited to solid tumors. These limitations led to the development of active targeting.

#### 2.1.1. Actively Targeted Liposomes

The active targeting approach enhances the targeting efficiency of liposomal carriers, improving therapeutic outcomes and minimizing the potential toxicity. One of the first active targeting approaches explored involves the use of liposomes integrated with magnetic nanoparticles. By applying an external magnetic field, these magnetized liposomes can be precisely directed to tumor sites, enhancing the targeting efficiency and improving therapeutic outcomes while reducing the impact on healthy tissues [21]. An example of this approach is the use of ferri-liposomes, which encapsulate ferrimagnetic iron oxide nanoparticles [22]. These liposomes, when exposed to a magnetic field, can target tumors effectively, slow tumor growth in mouse models, and offer simultaneous drug delivery and MRI-based detection.

A classic active targeting strategy involves attaching targeting ligands to the liposomal surface, thus enhancing the delivery of the liposomal systems. Various molecules have been used as targeting ligands: antibodies, nucleic acids, peptides, proteins, and small molecules [23]. Several aspects are considered when selecting a targeting ligand: (i) the overexpression or selective expression of the target in the targeted tissue, (ii) the uptake of the targeted formulation by the target cells, and (iii) the degree of covering of the target molecule [24]. The most commonly used targeting agents are antibodies; liposomes functionalized with antibodies are termed immuno-liposomes [25,26,27]. Monoclonal antibodies against receptors, such as HER2 [28], EGFR [29,30], and transferrin receptor [31], among others, have been successfully used to prepare immuno-liposomes. Some of these formulations have the added benefit of blocking the targeted receptor, resulting in increased anti-cancer activity [28,32]. Efforts in developing cancer-targeting liposomes have focused not only on cancer cells, but also on the tumor microenvironment; the targets include various overexpressed proteases, such as cathepsins [33,34,35] or MMPs, vascular cell adhesion molecules [36], and integrins [37,38].

**Figure 1:**
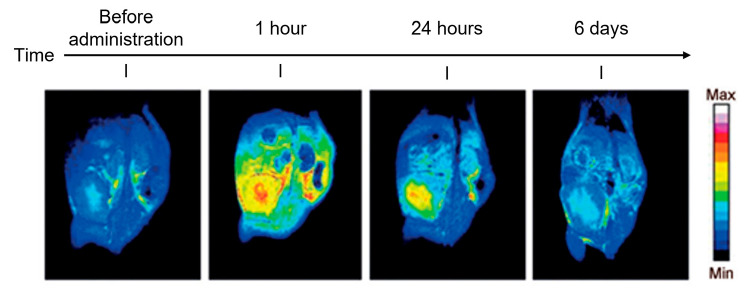
The tumor targeting of the MRI contrast agent Magnevist by LNC-NS-629 in a mouse breast cancer model. Adapted with permission from Mikhaylov et al. (2014) [33].

One of the very first applications of a liposomal nanocarrier attached to a small molecule targeting a tumor-associated protease, cathepsin B (CtsB), was described by Mikhaylov et al. in 2014 [33]. A selective CtsB inhibitor conjugated to a PEG-functionalized lipid was incorporated into the envelope of a liposomal nanocarrier. The resulted targeted drug delivery system, LNC-NS-629, was demonstrated to be efficient in targeting a clinical MRI contrast agent to the tumor in a mouse breast cancer model (Figure 1).

The efforts to target cysteine cathepsins were further pursued by functionalizing liposomes with the endogenous cysteine cathepsins inhibitor stefin A [34]. The novel delivery system was shown to selectively target cathepsins in vitro and in vivo. In an additional study, the aspartic protease cathepsin D was targeted using pepstatin A—its natural peptide inhibitor [35]. The pepstatin A-functionalized liposomes were efficient in targeting cathepsin D in cancer cell lines, showing potential for their use in vivo.

Although the concept of active targeting is very promising and numerous studies have focused on developing novel actively targeted systems, there are some limitations, among them the higher development costs for targeted liposomes; this might be the reason progress to the clinic has been slow [39].

#### 2.1.2. Triggered Liposomes

The stability of liposomes in circulation and the retention of their contents are key characteristics for a successful delivery system. Efforts have therefore been employed in the development of triggered liposomes, which would protect the encapsulated drug during trafficking and release their contents upon reaching the target tissue. There are two major types of triggers used to produce this effect: remote triggers, such as heat, ultrasound, and light, and local triggers intrinsic to the targeted tissue, such as enzymes and pH distortions [40]. The exploitation of these triggers has resulted in the development of several advanced technologies, such as thermosensitive liposomes [41,42,43], light-sensitive liposomes [44,45,46], echogenic liposomes triggered by ultrasound [47], enzyme-responsive liposomes [48,49,50], and pH-sensitive liposomes [51].

The first generation of triggered liposomes employed thermosensitivity to release the encapsulated drug in response to temperature fluctuations [42,43] that could be achieved by the application of an external heating source, such as a water bath, laser, or electromagnet [52]. Thermosensitive liposomes were initially developed by adjusting the lipid bilayer components to achieve a transition temperature (Tm) around 41 °C, at which point the bilayer shifts from a solid gel phase to a liquid crystalline phase, triggering the release of the encapsulated compounds [53]. Newer developments led to the inclusion of thermosensitive polymers, offering more tunability [54]. Several thermosensitive liposome formulations have been evaluated in clinical trials. One of the leading clinical trials of ThermoDox^®^, a lyso-thermosensitive liposomal doxorubicin (LTLD), has progressed to the phase III stage, with two trials completed [55]. The initial phase III clinical trial known as the HEAT trial evaluated the combination of ThermoDox^®^ with radiofrequency ablation (RFA) compared to RFA alone for the treatment of inoperable hepatocellular carcinoma (HCC). Although conducted on 701 patients across 55 centers globally, the trial did not meet its primary endpoint of progression-free survival (PFS). The subsequent phase III OPTIMA trial, including 556 patients who were treated with ThermoDox^®^ and underwent RFA for at least 45 min, did not show statistically significant improvement in the primary endpoint. While certain patient subgroups appeared to show clinical benefit, the company decided not to pursue these retrospective findings due to the significant regulatory challenges that could impact further development. Despite these setbacks, both trials provided valuable insights into the challenges of combining thermally activated drug delivery systems with RFA, highlighting the need for continued research to optimize treatment protocols, heating modalities, and other factors for better therapeutic outcomes [56].

Light-sensitive or photoactivatable liposomes incorporate components that respond to light in their structure. These molecules can be photosensitizers, which generate reactive oxygen species, or they can undergo a conformational change, cleavage, polymerization, or generate heat after absorbing light [57]. The changes in the molecules following exposure to light leads to the destabilization of the liposome membrane, resulting in the release of the drug cargo. The first photoresponsive liposome preparation was described in 1981 by Kano et al. Light as an activation stimulus offers high spatial and temporal control for drug release, while being non-invasive. However, some challenges still hinder the translation of this technology to clinics—low tissue penetration and concerns regarding UV phototoxicity [58]. An additional drawback, common to all remotely triggered liposomal release systems in cancer, is that they can only target primary tumors, while patients mostly die from metastatic disease [39].

pH-sensitive liposomes take advantage of the lowered extracellular pH in tumors, thus providing a local stimulus for drug release [59]. These liposomes are often composed of compounds that are protonated at an acidic pH and thus destabilize the liposomal membrane, or of compounds that undergo conformational changes at an acidic pH [60]. Newer strategies include pH-sensitive polymers incorporated into the liposomal membrane [61]. Like conventional liposomes, pH-sensitive liposomes also faced the problem of being eliminated from circulation too quickly; therefore, PEG coating was added to prolong their circulation time [62]. These liposomes are also internalized more efficiently, which might be beneficial for bypassing drug transporters in MDR2-overexpressing tumor cells, thus evading drug resistance [63].

Another approach using local stimuli is enzyme-responsive liposomes, which take advantage of the enzymes overexpressed in the tumor tissue [53]. Research has mostly been focused on liposomes triggered by phospholipases [49,64] and matrix metalloproteases [50,65]. To achieve activation by phospholipase, the lipids used need to be degradable by this enzyme, while for activation by MMPs, specialized lipopeptides that are substrates for the MMPs need to be utilized [66].

#### 2.1.3. Multifunctional Liposomes

**Figure 2:**
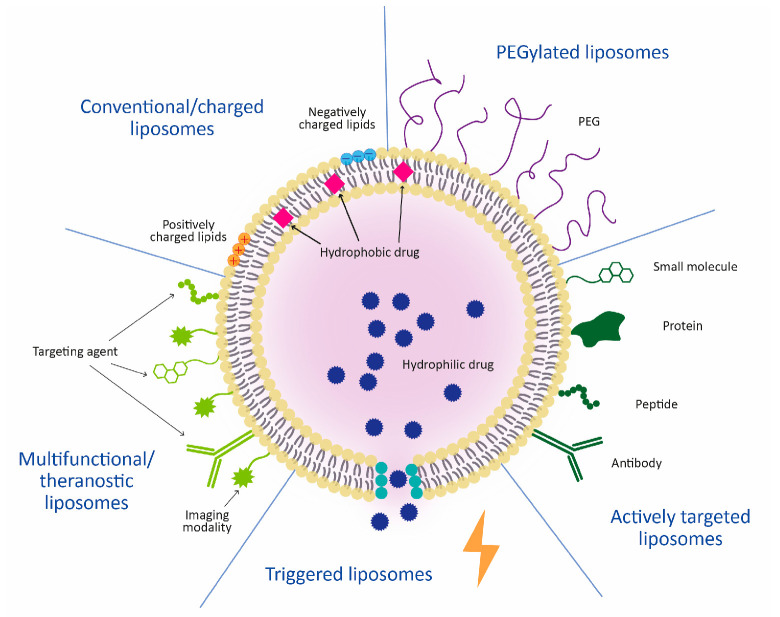
Different liposome types differing in their lipid composition or surface modifications.

Different features of liposomes can be combined to yield multifunctional liposomes (Figure 2). Theranostic liposomes include diagnostic and therapeutic functions and allow for the monitoring of real-time delivery, easier determination of therapy responses, and more accurate diagnoses [67]. Theranostic liposomes should include an imaging modality, which can be an optical dye for fluorescence imaging [68] or a contrast agent for magnetic resonance imaging (MRI) [69], computed tomography (CT) [70], positron emission tomography (PET) [71], single-photon emission computed tomography (SPECT) [72], and photoacoustic imaging [73], or even a combination of multiple modalities [74].

Theranostic liposomes have been mostly developed for cancer therapy and thus combine the imaging modality with anti-cancer drugs, which can be chemotherapeutics or various sensitizing agents. Often, targeting molecules are also added to the theranostic system. A few examples of this technology include ferri-liposomes loaded with iron oxide for MRI and magnetic targeting as well as doxorubicin [22], TME-targeted liposomes loaded with Magnevist for MRI and doxorubicin [33], nano-liposomes encapsulating the contrast agent iodixanol and photosensitizers for CT imaging-guided phototherapy of cervical cancer [75], iron oxide liposomes loaded with doxorubicin [76], gold-containing liposomes with radioisotopes for breast cancer photothermal therapy combined with PET imaging [77], transferrin receptor-targeted gold-based liposomes containing the chemotherapeutic docetaxel for brain-targeted drug delivery and imaging [78], and folate-targeted liposomes encapsulating paclitaxel and vinorelbine for anti-cancer activity as well as the radioisotope Tc-99m for SPECT/CT imaging [79]. These studies demonstrate the versatility of the liposomal platforms and their potential in combining many different modalities, potentially leading to more effective combination therapies.

Another approach to label liposomes can be achieved by the incorporation of fluorescent lipids. First, they can trigger rapid membrane fusion between cellular plasma membranes and the lipid bilayers of their carrier particles, so-called fusogenic liposomes, and second, after insertion into the cellular membranes, they can enable fluorescence imaging of the traffic processes and fusion with the cell membranes [80].

### 2.2. Liposome Preparation and Characterization

**Figure 3:**
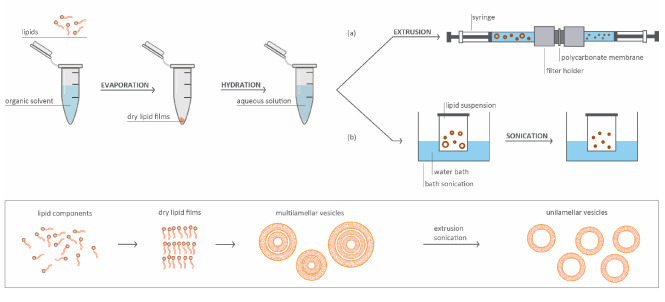
A schematic diagram of liposome preparation. Lipid components dissolved in an organic solvent are dried to form thin lipid films, which are then hydrated with an aqueous solution to produce multilamellar vesicles. (**a**) Extrusion through a polycarbonate membrane to form unilamellar vesicles with a uniform size. (**b**) Sonication using a water bath to break down multilamellar vesicles into smaller unilamellar vesicles.

The first developed and still the most common method used to prepare liposomes was thin-film hydration, where all lipids and other hydrophobic components are dissolved in a suitable organic solvent. The solvent is then gently evaporated and the obtained thin film is hydrated with an aqueous buffer solution [81] (Figure 3). Other traditional methods for liposome preparation include reverse-phase evaporation [82], ethanol injection [83], high-pressure homogenization, the freeze–thaw method, detergent removal, and others [82]. Most of these methods produce MLVs or LUVs; if smaller unilamellar vesicles need to be prepared, the next step is often sonication or extrusion through a polycarbonate membrane [84]. Drug loading is another crucial step in liposome preparation and can be done passively or actively. Passive loading entraps hydrophilic molecules in the aqueous core during lipid bilayer formation, while hydrophobic molecules are dried together with the lipids and thus incorporated in the lipid bilayer. Passive loading has some major drawbacks, such as drug leakage, low encapsulation efficiency, and bilayer destabilization [84]. To improve the efficiency of drug loading, active or remote loading has been developed. This approach relies on the creation of an ionic or pH gradient across the liposomal membrane. Uncharged drugs are added to preformed liposomes and can cross the membrane, but once they enter the liposomal core, they become protonated and can no longer pass the bilayer. However, this approach works only for some compounds, ideally for amphipathic weak bases or weak acids [85].

After production and before application, liposomes need to be well evaluated for their physical and chemical properties to ensure their performance. The size and the polydispersity index (PDI) are the most relevant features in liposome characterization. It has been shown that size influences the circulation time of liposomes, with large liposomes being eliminated more quickly [86]. For drug delivery, liposomes sized between 50 and 200 nm are considered ideal. The PDI value refers to the heterogeneity of the liposomes’ sizes in the sample. A lower value indicates a more uniform sample, which is more suitable for drug delivery applications [87]. The most common method used to measure liposomal size is dynamic light scattering (DLS), which correlates the amount of scattered light to the mean size of the liposomes. The surface charge of liposomes affects not only their stability in solution, but also their adsorption into tissue, protein binding, and opsonization. Thus, the overall net charge of the particles, usually expressed as the surface or zeta potential, is another key feature of liposomal characterization. Nanoparticles with a zeta potential of at least 30 mV are considered stable, as they are charged enough to prevent aggregation. Additionally, higher zeta potential values lead to stronger membrane bindings and higher levels of cellular uptake [88]. DLS can be employed to measure the zeta potential [89].

In terms of drug loading and retention, encapsulation efficiency (EE) and in vitro drug release are the relevant characteristics. The EE is calculated as the percentage of the amount of the drug inside the liposomes compared to the total amount of the drug used in the liposome preparation. To determine the EE, the non-encapsulated free drug must first be separated from the liposomes. This can be achieved using various methods, including size exclusion chromatography, centrifugation or ultracentrifugation, and dialysis membrane with an appropriate cut-off. The next step is determining the amount of the encapsulated drug, either directly by measuring the amount of the drug in the liposomes or indirectly by measuring the free drug and subtracting it from the total drug used. Methods for assessing the concentration of the drug depend mostly on the drug molecule and range from UV–Vis or fluorescence spectroscopy and protein-based assays to mass spectrometry [90]. The in vitro drug release profile refers to the cumulative release percentage over time. It is measured using dialysis conditions—the liposomes are placed in a dialysis bag membrane with a specific molecular cut-off. The bag is placed into a simulated physiological fluid kept at 37 °C to mimic an in vivo environment. At selected time points, the concentration of the released drug is measured [91].

### 2.3. Cellular Internalization Mechanisms

The cellular internalization of liposomes is a complex process influenced by various factors, including liposome composition, size, charge, and the specific endocytic pathways utilized by the target cells. Liposomes can enter cells through several mechanisms, primarily endocytosis, which includes clathrin-mediated endocytosis, caveolae-mediated endocytosis, and micropinocytosis [92,93,94].

Cationic liposomes, which carry a positive charge, have been shown to enhance cellular uptake due to their favorable interactions with the negatively charged cell membranes. It was demonstrated that cationic liposomes effectively internalize into neutrophils via natural phagocytosis, facilitated by their positive zeta potential, which enhances the membrane interactions [95]. Similarly, the work by Mudhakir et al. indicated that cationic liposomes are internalized efficiently through clathrin-mediated and caveolar endocytosis, highlighting the importance of the liposome charge in determining the uptake pathway [96]. The size of the liposomes also plays a critical role in their internalization efficiency. Research by Sakai-Kato et al. indicated that liposomes with diameters around 100 nm are particularly effective for cellular uptake [97]. This observation is supported by further findings that the diameter of the liposomes significantly influences their endocytic uptake, suggesting an optimal size for effective internalization [98]. Furthermore, the functionalization of liposomes with targeting ligands can enhance their uptake through receptor-mediated endocytosis. Sun et al. illustrated that folate-functionalized liposomes bind to folate receptors on target cells, leading to efficient internalization via endosomal pathways [99]. This targeted approach is further supported by the work of He et al., which demonstrated that gonadorelin-modified liposomes achieved a significantly higher uptake in target cells through specific receptor-mediated endocytosis than nonmodified liposomes [100]. In addition to charge and size, the physicochemical properties of liposomes, such as membrane fluidity and rigidity, also affect their internalization. It was shown that increased membrane fluidity could enhance the endocytosis of phosphatidylserine-containing liposomes, while rigid liposomes may require more energy for effective internalization [101]. This is consistent with findings from Takechi-Haraya et al., which emphasized the importance of liposome membrane characteristics in facilitating liposome uptake [102].

In summary, the internalization of liposomes into cells is a multifaceted process influenced by their charges, sizes, surface modifications, and physicochemical properties. Understanding these factors is crucial for optimizing liposome design for targeted drug delivery applications.

## 3. Clinical Applications

Liposomes offer a compelling approach for drug delivery systems, particularly in oncology, where they can contribute to improved drug stability, targeted delivery, controlled release, and reduced toxicity. In recent years, several liposomal formulations have successfully progressed from pre-clinical research to the clinic, offering promising solutions for targeted drug delivery. This section highlights the liposomal-based therapies that have received regulatory approval (Table 1) and those currently undergoing clinical trials (Table 2). Notably, the described ongoing clinical trials evaluate new liposomal formulations for applications in chemotherapy, immunotherapy, and other treatment modalities, emphasizing their adaptability to a variety of medical treatments. By reviewing these developments and clinical experiences, we can better understand the clinical applicability of the liposomal formulations and identify the challenges that remain in advancing their use. Taken together, these developments reflect the growing confidence in liposomal technology, with the ongoing trials playing a key role in advancing their broader clinical applications.

### 3.1. Approved Liposomal Therapeutics

The first liposomal formulation approved for clinical use was Doxil^®^ in 1995 (named Caelyx^®^ in Europe) [7]. Doxil is a formulation of liposomes containing the cytostatic drug doxorubicin hydrochloride. Doxil liposomes are composed of hydrogenated soy phosphatidylcholine (HSPC), cholesterol, and lipidated PEG. This composition results in a high phase transition temperature, forming a non-flexible bilayer at 37 °C. The liposomal surface is covered with PEG molecules, ensuring a longer circulation time [103]. Doxorubicin loading is performed actively using a transmembrane gradient of ammonium sulfate, which results in more than 90% EE [7]. Doxil was at first approved for AIDS-related Kaposi’s sarcoma, and later for recurrent ovarian cancer, metastatic breast cancer, and multiple myeloma. Doxil has shown superior clinical performance compared to free doxorubicin and is still one of the most extensively used liposomal drugs; it accumulates more selectively in tumor tissue, minimizing the cardiotoxic effect of doxorubicin. The levels of accumulated doxorubicin were 4 to 16 times higher compared to the free doxorubicin administration. Additionally, the drug levels at the tumor tissue peaked between 3 to 7 days after administration [104]. In spite of the overall superior tolerability of Doxil, two side effects not typical in the use of the free drug were observed. The first one is Palmar Plantar Erythrodysthesia (PPE), which shows up as redness, tenderness, and peeling of the skin and limits the dose of Doxil [105]. The second is a complement activation-related pseudo-allergy (CARPA), an acute hypersensitivity or infusion reaction, which many nano-systems can provoke [106].

Other liposomal formulations encapsulating similar anti-cancer drugs were developed soon after. In 1996, DaunoXome^®^ was approved for the treatment of HIV-associated Kaposi’s sarcoma with a formulation composed of non-PEGylated liposomes containing daunorubicin, a drug from the same family—anthracyclines—as doxorubicin [107]. Similar to Doxil, DaunoXome also reduced cardiotoxicity and improved efficacy compared to the free drug. However, the response rates of Doxil are higher than those of DaunoXome, potentially due to Doxil’s PEGylation resulting in longer circulation times [108]. Myocet^®^ is a formulation of doxorubicin encapsulated in non-PEGylated liposomes, which leads to a reduced incidence of PPE, but also to more rapid clearance of the liposomes. It was approved for metastatic breast cancer in 2000 in Europe [109].

In the case of Mepact^®^, approved in 2004 for non-metastatic osteosarcoma, uptake by the mononuclear phagocyte system was not an unwanted effect. Mepact is a liposomal form of muramyl tripeptide phosphatidyl ethanolamine (MTP-PE), which is an immunostimulating compound; it activates macrophages to become tumoricidal. Compared to the free MTP-PE, the liposomal form was more efficient at activating the monocytes, was retained longer in the target organs, and was less toxic due to its fast clearance from circulation by the phagocytic cells of the reticuloendothelial system, and thus a lower systemic exposure [110].

Marqibo^®^ was designed to overcome the sub-optimal pharmacokinetics and dose-related neurotoxicity of vincristine, a highly active cell cycle-dependent anti-cancer drug. The liposomal form achieved a longer plasma circulation time, increased tumor tissue delivery and accumulation, and slowed the release of vincristine in the tumor tissues. These features translated to higher efficacy and tolerability compared to the free vincristine [111]. Marqibo was approved by the FDA in 2012 for the treatment of leukemia.

Similar benefits were observed with Onyvide^®^, the liposomal form of irinotecan. Irinotecan is an anti-cancer drug with a broad spectrum of activity and DNA topoisomerase 1 as its primary target. Irinotecan’s pharmacology is complex and multistep, requiring activation of the prodrug by the liver enzymes [112]. The liposomal encapsulation resulted in a longer circulation time and prolonged tumor exposure, leading to a smaller dose of the liposomal drug needed to achieve similar effects compared to the free drug [113].

Vyxeos^®^ is a formulation of cytarabine and daunorubicin at a 5:1 ratio. This ratio was pre-clinically determined to be optimally synergistic for this drug combination. When free drugs are injected, their initial ratios change within minutes and the synergistic effect might be diminished. The liposomal encapsulation ensures fixed-ratio drug delivery to the target tumor tissue, in addition to other advantages of the liposomal systems [114]. Treatment with Vyxeos was associated with longer survival compared to the standard-of-care cytarabine plus daunorubicin chemotherapy [115]. Vyxeos was approved for the treatment of acute myeloid leukemia in 2017.

In contrast to other marketed liposomal anti-cancer drugs, Depocyt^®^ is composed of multivesicular liposomes, which are associated with higher stability and slower drug release compared to standard unilamellar vesicles. Depocyt was approved in 1999 for the treatment of neoplastic meningitis. A significant obstacle in the treatment of neoplastic meningitis is the poor penetration of systemically administered chemotherapeutics into the cerebrospinal fluid. Intrathecal injection is thus the preferred treatment; however, the half-life of cytarabine is short, while its mechanism demands prolonged exposure. A sustained release formulation using multivesicular liposomes was developed to overcome this. Treatment with Depocyt was associated with a longer time of survival and fewer adverse side effects, with an additional advantage of less frequent dosing [116].

### 3.2. Selected Liposomal Formulations in Clinical Trials

Numerous novel liposome formulation-based therapies for cancer treatment have been developed and are undergoing clinical trials for the evaluation of their safety, dosage, and effectiveness, with applications spanning chemotherapy, immunotherapy, and radiotherapy, among many others (Table 2).

The first approved Doxil^®^/Caelyx^®^ lipid formulation, composed of hydrogenated soybean phosphatidylcholine (HSPC), 1,2-distearoyl-sn-glycero-3-phosphoethanolamine-N-[methoxy(polyethylene glycol)] (MPEG2000-DSPE), and cholesterol, is currently being evaluated in several phase II and III clinical trials for treating a variety of cancers, including triple-negative breast cancer (clinicaltrials.gov, NCT03971409) and recurrent ovarian cancer (clinicaltrials.gov, NCT06014528, NCT02839707, NCT00326456), in combination with agents such as avelumab, binimetinib, atezolizumab, and carboplatin. Different formulations of encapsulated doxorubicin have been developed over the years, with significant modifications made to the lipid composition, thermostability, liposomal size, and charge, achieving superior drug entrapment efficiencies, prolonged stable storage, higher release performance and circulation times, and reduced uptake by vital organs [117]. For instance, Talidox^®^, a new liposomal doxorubicin formulation currently undergoing a clinical trial in advanced solid tumors (clinicaltrials.gov, NCT03387917), has been designed with a particle size twice as small as conventional liposomal formulations, thus enabling deeper penetration into tumors [118].

Besides doxorubicin, other chemotherapeutic drugs have been applied to liposomal formulation and drug delivery. Topotecan, a small molecule drug and topoisomerase inhibitor encapsulated in a liposomal formulation [119] composed of dihydrosphingomyelin (DHSM), cholesterol, and polyethylene glycol (FF-10850), is undergoing a phase I trial (clinicaltrials.gov, NCT04047251) to assess its safety in treating advanced solid tumors such as Merkel cell carcinoma [120]. Irinotecan, another topoisomerase inhibitor [119], is encapsulated in the Nal-IRI (Onivyde^®^) liposomal formulation, composed of 1,2-distearoyl-sn-glycero-3-phosphocholine (DSPC), cholesterol, and MPEG2000-DSPE [121] and currently undergoing a phase III trial for advanced pancreatic cancer (clinicaltrials.gov, NCT03468335). This formulation is being tested in combination with fluorouracil and leucovorin as a second-line therapy. Additionally, CPX-351 (Vyxeos^®^) is a phase II liposomal formulation of DSPC, 1,2-distearoyl-sn-glycero-3-phosphoglycerol (DSPG), and cholesterol. These liposomes encapsulate two chemotherapeutic agents, daunorubicin and cytarabine (clinicaltrials.gov, NCT04269213), and are being tested for the treatment of secondary acute myeloid leukemia in patients younger than 60 years old, both as a monotherapy and in combination with ruxolitinib [122].

In parallel with small molecule drug delivery, liposomal nanoparticles also show great promise in cancer immunotherapy, including vaccines. For instance, nanoparticles enhance the effectiveness of cancer vaccines by ensuring prolonged persistence in tissues as well as the controlled release of antigens and adjuvants [123]. One such example is the autologous tumor mRNA-loaded liposome vaccine, composed of cationic lipid DOTAP, which has been studied for its ability to induce immune responses against melanoma (clinicaltrials.gov, NCT05264974) [124]. In addition to their role in cancer vaccines, liposomal formulations are increasingly used in cancer immunotherapy to enhance drug delivery, modulate the immune response, and improve the therapeutic efficacy of approaches such as immune checkpoint blockade and cancer vaccines [125]. In line with this, HF1K16 is a phase I investigational liposomal formulation of all-trans retinoic acid (ATRA) utilizing HSPC and MPEG-DSPE, designed for sustained drug release to target solid tumors by promoting MDSC maturation and modifying the tumor microenvironment to enhance immune responses (clinicaltrials.gov, NCT05388487) [126].

Liposomal formulations are also used to encapsulate antisense oligonucleotides (ASOs) for cancer treatment, as they facilitate efficient drug distribution, improve cellular uptake, and allow the ASOs to bypass the endocytic degradation process, ensuring a more effective delivery to the target tumor cells [127]. In this context, BP1002, a phase I liposomal formulation containing an antisense oligonucleotide targeting the Bcl-2 transcript (clinicaltrials.gov, NCT05190471), is being tested for the treatment of refractory or relapsed acute myeloid leukemia. The critical role of the pro-survival and pro-apoptotic Bcl-2 family oncoproteins in the regulation of apoptosis makes them attractive targets for the treatment of cancer [128]. The BP1002 liposomes, formed from 1,2-dioleoyl-sn-glycero-3-phosphocholine (DOPC), are being evaluated in combination with venetoclax and decitabine to improve future treatment outcomes [129].

These examples demonstrate the continued exploration and potential of liposomal therapies to enhance drug delivery, reduce side effects, and improve efficacy in treating both solid tumors and hematological malignancies.

**Table 1:** Liposomal formulations approved for clinics.

Product Name	Approval Year	Active Agent	Lipid Composition	Injection Route	Indication	Company
Doxil^®^ (US) Caelyx^®^ (EU)	1995 (US) 1996 (EU)	Doxorubicin	MPEG-DSPE, HSPC, cholesterol	i.v.	Metastatic ovarian carcinoma, AIDS-related Kaposi’s sarcoma, multiple myeloma, breast cancer	Baxter Healthcare Corporation
DaunoXome^®^	1996 (US)	Daunorubicin	DSPC, cholesterol	i.v.	AIDS-related Kaposi’s sarcoma	NexStar Pharmaceuticals
DepoCyt^®^ (US, EU)	1999 (US) 2001 (EU)	Cytarabine	Cholesterol, triolein, DOPC, DPPG	Intrathecal	Lymphomatous meningitis	Pacira Pharmaceuticals
Myocet^®^	2000 (EU)	Doxorubicin	Phosphatidylcholine, cholesterol	i.v.	Metastatic breast cancer	Cheplapharm Arzneimittel
Mepact^®^	2009 (EU)	MTP-PE	POPC, OOPS	i.v.	Non-metastatic osteosarcoma	Takeda France
Marqibo^®^	2012 (US)	Vincristine sulfate	Sphingomyelin, cholesterol	i.v.	Acute lymphoblastic leukemia	Talon Pharmaceuticals
Onivyde^®^	2015 (US) 2016 (EU)	Irinotecan	DSPC, cholesterol, MPEG2000-DSPE	i.v.	Metastatic pancreatic adenocarcinoma	Merrimack Pharmaceuticals
Vyxeos^®^	2017 (US) 2018 (EU)	Daunorubicin, cytarabine	DSPC, DSPG, cholesterol	i.v.	Acute myeloid leukemia	Celator Pharmaceuticals

DOPC: 1,2-dioleoyl-sn-glycero-3-phosphocholine; DPPG: dipalmitoylphosphatidylglycerol; DSPC: 1,2-distearoyl-sn-glycero-3-phosphocholine; DSPG: 1,2-distearoyl-sn-glycero-3-phosphoglycerol; HSPC: hydrogenated soybean phosphatidylcholine; MPEG-DSPE: 1,2-distearoyl-sn-glycero-3-phosphoethanolamine-N-[methoxy(polyethylene glycol)]; MPEG2000-DSPE: 1,2-distearoyl-sn-glycero-3-phosphoethanolamine-N-[methoxy(polyethylene glycol)-2000]; OOPS: 1,2-dioleoyl-sn-glycero-3-phospho-L-serine monosodium salt; POPC: 1-palmitoyl-2-oleoyl-sn-glycero-3-phosphocholine.

**Table 2:** Liposomes under different phases of clinical trials.

Product Name	Active Drug	Lipid Composition	Phase	Indication	Therapy Type	Sponsor	NTC Number
FF-10850	Topotecan	DHSM, cholesterol, polyethylene glycol	I	Advanced solid tumors (Merkel cell carcinoma)	Monotherapy	Fujifilm Pharmaceuticals U.S.A., Inc.	NCT04047251 [120]
FF-10832	Gemcitabine	Cholesterol, HSPC, N-MPEG-DSPE (4:15:1) [130]	I	Advanced solid tumors, biliary tract cancer	Monotherapy	Fujifilm Pharmaceuticals U.S.A., Inc.	NCT03440450 [131]
FF-10832	Gemcitabine	Cholesterol, HSPC, MPEG-DSPE (4:15:1) [130]	II	Urothelial and non-small cell lung cancer	Combination with pembrolizumab	Fujifilm Pharmaceuticals U.S.A., Inc.	NCT05318573 [132]
186RNL	Rhenium-186	DSPC, cholesterol, (55:45) [133]	I/II	Malignant glioma	Monotherapy	Plus Therapeutics	NCT01906385 [134]
TLD-1 (Talidox^®^)	Doxorubicin	DSPC, cholesterol, MPEG-DSPE (58:37:5)	I	Solid tumors	Monotherapy	Swiss Group for Clinical Cancer Research	NCT03387917 [118]
THE001 (DPPG2-TSL-DOX)	Doxorubicin	DPPG	I	Soft tissue sarcoma	Combined with hyperthermia	Thermosome GmbH	NCT05858710
Doxil^®^/Caelyx^®^	Doxorubicin	HSPC, MPEG2000-DSPE, cholesterol (56.2:38.3:5) [135]	II	Triple-negative breast cancer	Combination with avelumab and/or without binimetinib	Hope Rugo, MD	NCT03971409
Doxil^®^/Caelyx^®^	Doxorubicin	HSPC, MPEG2000-DSPE, cholesterol (56.2:38.3:5) [135]	II	Recurrent ovarian cancer	In combination with IN10018	Inxmed (Shanghai) Co., Ltd.	NCT06014528
Doxil^®^/Caelyx^®^	Doxorubicin	HSPC, MPEG2000-DSPE, cholesterol (56.2:38.3:5) [135]	II/III	Recurrent ovarian, peritoneal cancer	Combination with atezolizumab and/or bevacizumab	National Cancer Institute (NCI)	NCT02839707
Doxil^®^/Caelyx^®^	Doxorubicin	HSPC, MPEG2000-DSPE, cholesterol (56.2:38.3:5) [135]	III	Ovarian cancer	Combination with carboplatin	National Cancer Institute, Naples	NCT00326456
Thermo-Dox^®^	Doxorubicin	DPPC, MSPC, MEG2000-DSPE (86:10:4) [136]	II	Relapsed solid tumors	Combination with hyperthermia	Children’s National Research Institute	NCT04791228
HF1K16	All-trans retinoic acid (ATRA)	HSPC, MPEG2000-DSPE [137]	I	Solid tumors	Monotherapy	Highfield Biopharmaceuticals Corporation	NCT05388487 [126]
CPX-351 (Vyxeos^®^)	Daunorubicin and cytarabine	DSPC, DSPG, cholesterol (7:2:1)	I/II	Advanced-phase myeloproliferative neoplasms	Monotherapy	Ohio State University Comprehensive Cancer Center	NCT03878199 [115]
CPX-351 (Vyxeos^®^)	Daunorubicin and cytarabine	DSPC, DSPG, cholesterol (7:2:1)	II	Secondary acute myeloid leukemia	Combination with ruxolitinib	Roswell Park Cancer Institute	NCT04269213 [122]
Nal-IRI (Onivyde^®^)	Irinotecan	DSPC, cholesterol, MPEG2000-DSPE (3:2:0,015) [121]	II	Metastatic pancreatic cancer	Monotherapy	ECOG-ACRIN Cancer Research Group	NCT04233866 [138]
Nal-IRI (Onivyde^®^)	Irinotecan	DSPC, cholesterol, MPEG2000-DSPE (3:2:0,015) [121]	III	Advanced pancreatic cancer	Combination with fluorouracil, leucovorin	AIO-Studien-gGmbH	NCT03468335
E7389-LF	Eribulin	HSPC, cholesterol, MPEG2000-DSPE [139]	I/II	Solid tumors	Monotherapy and in combination with nivolumab	Eisai Co., Ltd.	NCT03207672 NCT04078295 [140,141]
Mitoxantrone liposome	Mitoxantrone hydrochloride	HSPC, cholesterol, MPEG-DSPE	II	Metastatic nasopharyngeal carcinoma	In combination with PD-1 blockade	Ming-Yuan Chen	NCT06472713
Mitoxantrone liposome	Mitoxantrone hydrochloride	HSPC, cholesterol, MPEG-DSPE	III	Metastatic nasopharyngeal carcinoma	Combination with capecitabine	CSPC Zhongnuo Pharmaceutical Co., Ltd.	NCT05717764
L-ANN	Annamycin	NDLC	I/II	Acute myeloid leukemia	Monotherapy	Moleculin Biotech, Inc.	NCT05319587 [142]
SMP-3124LP	CHK1 (checkpoint kinase 1) inhibitor	NDLC	I/II	Advanced solid tumors	In combination with cytarabine	Sumitomo Pharma America, Inc.	NCT06526819 [143]
L-TC	Trans-crocetin	HSPC, cholesterol, MPEG2000-DSPE (6:2:1) [144]	III	Glioblastoma	Monotherapy	Institut de cancérologie Strasbourg Europe	NCT06477939
BP1001 BP1002	Antisense oligodeoxynucleotide against GRB2	DOPC [129]	II	Acute myeloid leukemia	Monotherapy and in combination with ventoclax, decitabine	Bio-Path Holdings, Inc.	NCT02781883 NCT05190471 [145]
Promitil^®^	Mitomycin-C	HSPC, cholesterol, MPEG2000-DSPE (11:6:1) [146]	II	Pancreatic and ovarian cancer	Monotherapy and in combination with decitabine	Lipomedix Pharmaceuticals Inc.	NCT06478862
Visudyne^®^	Verteporfin	Egg PG, DMPC, ascorbyl palmitate, butylated hydroxytoluene	I	Glioblastoma, recurrent glioblastoma	Monotherapy	William Read, Emory University	NCT04590664 [147]
LC (Lipo-Curc^®^)	Curcumin	DMPC (72 mg/mL), DMPG (8 mg/mL) [148]	I/II	Glioblastoma	Monotherapy	Signpath Pharma, Inc.	NCT05768919 [149]
PDS0101	HPV-targeted immunotherapy	DOTAP	I/II	Papillomavirus-associated oropharynx cancer	Combination with radiotherapy and temozolomide	Mayo Clinic	NCT05232851 [150]
Tumor mRNA liposome vaccine	pp65 full length LAMP1 mRNA	DOTAP	I	Melanoma	Combination with capecitabine	University of Florida	NCT05264974 [124]

DHSM: dihydrosphingomyelin; DMPG: 1,2-dimyristoyl-sn-glycero-3-phosphoglycerol; DMPC: 1,2-dimyristoyl-sn-glycero-3-phosphocholine; DOPC: 1,2-dioleoyl-sn-glycero-3-phosphocholine; PG: phosphatidylglycerol; DPPG: 1,2-dipalmitoyl-sn-glycero-3-phosphor-(10 -rac-glycerol); DSPC: 1,2-distearoyl-sn-glycero-3-phosphocholine; DPPC: 1,2-dipalmitoyl-sn-glycero-3-phosphocholine; DSPG: 1,2-distearoyl-sn-glycero-3-phosphoglycerol; HSPC: hydrogenated soybean phosphatidylcholine; MPEG-DSPE: 1,2-distearoyl-sn-glycero-3-phosphoethanolamine-N-[methoxy(polyethylene glycol)]; MSPC: 1-stearoyl-2-hydroxy-sn-glycero-3-phosphatidylcholine; MPEG2000-DSPE: 1,2-distearoyl-sn-glycero-3-phosphoethanolamine-N-[methoxy(polyethylene glycol)-2000]; NDLC: not defined lipid composition. The lipid compositions are provided as molar ratios.

## 4. Conclusions

In summary, liposome-based targeted drug delivery systems mark a significant advancement in pharmaceutical science, offering distinct advantages that enhance both the efficacy and safety of various therapies. Their biocompatibility and ability to encapsulate diverse drug types, both hydrophilic and hydrophobic, make liposomes highly versatile carriers in clinical settings. This review underscores how liposomal formulations have improved drug pharmacokinetics while reducing side effects, particularly in oncology, where targeted delivery specifically to the tumor cells is crucial.

Multiple clinical trials have solidified the role of liposomes in modern medicine, with several formulations receiving regulatory approval and becoming vital components of established treatment protocols. These advancements highlight not only the promise of liposomes across multiple therapeutic approaches as monotherapies, but also their potential for synergistic approaches with other standard-of-care approved drugs in combination therapies.

Although liposomal drug delivery systems hold significant promise for clinical use, there are several challenges preventing their more extensive implementation and regulatory approval. One key issue is the complexity and variability in the manufacturing process, which may require additional efforts to optimize the methods and ensure consistent product quality and performance. Regulatory agencies require rigorous, reproducible data to demonstrate safety, efficacy, and quality, but the complexity of liposome production often makes it challenging to meet these standards, requiring continuous optimization for consistency [151]. While liposomes offer targeted delivery, their behavior in the body can be unpredictable, with factors like size, lipid composition, surface charge, and targeting and release mechanisms of actions influencing their effectiveness. Additionally, there is still limited knowledge about their long-term pharmacokinetics and potential immunogenicity, raising concerns about their safety over time. High production costs and the need for extensive clinical trials to confirm therapeutic benefits further complicate their use [56,151]. Addressing these issues requires ongoing advancements in liposome technology, clearer regulatory pathways, and a deeper understanding of their clinical behavior across different treatments.

Future research thus should prioritize advancements in the targeting mechanisms through innovative surface modifications and the exploration of novel drug combinations to address the complexities of multifactorial diseases. With a deeper understanding of how liposomes interact with biological systems and improvement of their biochemical properties, we expect to see exciting developments that can broaden their applications. Ultimately, liposome-based drug delivery systems have the potential to transform therapeutic approaches, paving the way for more effective and personalized treatments for patients.
